# Purine and pyrimidine metabolism: Convergent evidence on chronic antidepressant treatment response in mice and humans

**DOI:** 10.1038/srep35317

**Published:** 2016-10-12

**Authors:** Dong Ik Park, Carine Dournes, Inge Sillaber, Manfred Uhr, John M. Asara, Nils C. Gassen, Theo Rein, Marcus Ising, Christian Webhofer, Michaela D. Filiou, Marianne B. Müller, Christoph W. Turck

**Affiliations:** 1 Max Planck Institute of Psychiatry, Department of Translational Research in Psychiatry, 80804, Munich, Germany; 2 Max Planck Institute of Psychiatry, Department of Stress Neurobiology and Neurogenetics, 80804 Munich, Germany; 3 Phenoquest AG, 82152 Martinsried, Germany; 4 Max Planck Institute of Psychiatry, Department of Clinical Research, 80804 Munich, Germany; 5 Division of Signal Transduction, Beth Israel Deaconess Medical Center, and Department of Medicine, Harvard Medical School, Boston, MA 02115, USA.; 6 Experimental Psychiatry, Department of Psychiatry and Psychotherapy & Focus Program Translational Neuroscience, Johannes Gutenberg University Medical Center, 55128 Mainz, Germany

## Abstract

Selective Serotonin Reuptake Inhibitors (SSRIs) are commonly used drugs for the treatment of psychiatric diseases including major depressive disorder (MDD). For unknown reasons a substantial number of patients do not show any improvement during or after SSRI treatment. We treated DBA/2J mice for 28 days with paroxetine and assessed their behavioral response with the forced swim test (FST). Paroxetine-treated long-time floating (PLF) and paroxetine-treated short-time floating (PSF) groups were stratified as proxies for drug non-responder and responder mice, respectively. Proteomics and metabolomics profiles of PLF and PSF groups were acquired for the hippocampus and plasma to identify molecular pathways and biosignatures that stratify paroxetine-treated mouse sub-groups. The critical role of purine and pyrimidine metabolisms for chronic paroxetine treatment response in the mouse was further corroborated by pathway protein expression differences in both mice and patients that underwent chronic antidepressant treatment. The integrated -omics data indicate purine and pyrimidine metabolism pathway activity differences between PLF and PSF mice. Furthermore, the pathway protein levels in peripheral specimens strongly correlated with the antidepressant treatment response in patients. Our results suggest that chronic SSRI treatment differentially affects purine and pyrimidine metabolisms, which may explain the heterogeneous antidepressant treatment response and represents a potential biosignature.

Although Selective Serotonin Reuptake Inhibitors (SSRIs) have been used as preferred antidepressant medications for several decades, over one third of major depressive disorder (MDD) patients do not respond to SSRI treatment^1^. The high variability in response to SSRIs combined with a lack of clinically useful assessments that can reliably determine whether a patient responds to a particular antidepressant compound currently prevent a strategic treatment and personalized medicine approach in psychiatry.

The identification of genetic factors that could assist in the prediction and determination of an individual’s drug response has been a major focus in psychiatry research. However, despite great efforts in large genome-wide association studies, the results are fairly modest. Few genetic polymorphisms identified have achieved genome-wide significance or were consistently replicated across studies, suggesting that alternative strategies need to be explored to realize molecular stratification of antidepressant treatment response^2^,^3^. Both, biomarkers determining *a priori* whether an individual patient responds to the treatment of choice as well as a distinction of responders and non-responders during antidepressant therapy can have a significant impact to achieve this goal. Biosignatures including proteins and metabolites would not only allow to monitor treatment response in clinical practice, but also assist in the evaluation of drug actions at an early stage in clinical trials which are frequently marred by late attrition.

Recent findings obtained with clinical specimens indicate a potent role of metabolites for separating antidepressant responder and non-responder patients. Baseline plasma levels of 3-methoxy-4-hydroxyphenylglycol, a noradrenaline metabolite, were different between paroxetine responder and non-responder patients^4^. Several pathways involved in dihydroxyphenylacetic acid, serotonin, and gamma tocopherol synthesis have been implicated in separating sertraline responder and non-responder patients^5^. However, despite these promising findings with peripheral patient specimens an understanding of the molecular changes in response to SSRI treatment occurring in the brain is still missing. A systematic investigation of affected pathways in the brain and a correlation with the periphery will eventually allow the implementation of biosignatures capable of differentiating antidepressant responders and non-responders.

In the current study, a great number of inbred DBA/2J mice were treated for 28 days with a commonly used SSRI, paroxetine. The DBA/2J mouse strain was previously shown to be responsive to oral treatment with paroxetine under basal stress-free conditions^6^. Animals were stratified into long-time floating (PLF) and short-time floating (PSF) groups according to their forced swim test (FST) floating time. Metabolite profiles in the hippocampus and plasma of the PLF and PSF mice were assessed, and hippocampal protein profiles were integrated to identify pathways pertinent for the chronic paroxetine treatment response. We followed up our studies in the mouse with an analysis of specimens from patients that underwent chronic antidepressant treatment. We found that affected pathways identified in the mouse were also implicated in peripheral blood mononuclear cells (PBMCs) from antidepressant non-responder and responder patients.

## Results

### Sub-grouping of paroxetine responder and non-responder mice

**Figure 1:**
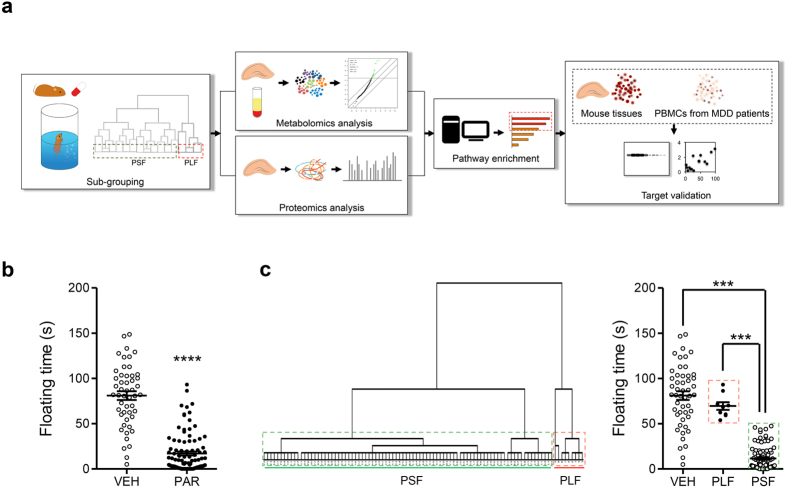
Sub-grouping of paroxetine-treated mice. (**a**) A schematic overview of the workflow. (**b**) Male DBA/2J mice received paroxetine (5 mg/kg, twice a day, 28 days) and floating time was recorded for 6 min. PAR mice displayed significantly shorter floating time compared to vehicle-treated group (VEH). *n*(VEH/PAR) = 50/95. (**c**) Dendrogram of paroxetine-treated mice. Mice treated with paroxetine were separated into PLF and PSF groups using hierarchical clustering analysis (HCA). *n*(VEH/PLF/PSF) = 50/9/86. Data are expressed as mean ± SEM. ***p < 0.001 (one-way ANOVA with Tukey’s test for multiple comparisons), ****p < 0.0001 (two-tailed *t*-test).

Figure 1a shows a schematic overview of the workflow. DBA/2J mice received vehicle or paroxetine pills (2 × 5 mg/kg/day) for 28 days. The paroxetine-treated group (PAR) showed significantly reduced FST floating time compared to vehicle-treated (VEH) mice (t = 13.90, df = 143, *p* < 0.0001) (Fig. 1b). We were able to separate paroxetine-treated mice into long-time floating (PLF) and short-time floating (PSF) groups according to their FST floating time using hierarchical cluster analysis (HCA) (F = 159.5, df = 144, *p* < 0.001) (Fig. 1c). PSF mice floating time was significantly lower than for VEH mice (*p* < 0.001) while no floating time difference was observed between PLF and VEH mice. During the female urine sniffing test (FUST) PLF and PSF mice did not show differential sniffing time prior to paroxetine treatment (F_(5,192)_ = 104.5, *p* > 0.05) (see Supplementary Fig. S1). A slightly different sniffing time became apparent after 28 days of paroxetine treatment (*p* = 0.052). The time for sniffing female urine was longer for PSF mice compared to VEH and PLF mice (F_(5,192)_ = 84.24, df = 197, *p* < 0.0001) (see Supplementary Fig. S1). Two-way ANOVA identified no interaction between time points and treatment.

### Covariates analysis

Covariates that may be relevant for paroxetine treatment response were examined. Paroxetine concentration in whole brain and plasma did not differ between groups (*p* > 0.05) and did not correlate with FST floating time (r = 0.26, *p* = 0.387) (see Supplementary Fig. S2). We also analyzed mouse age and body weight gain. A significant age effect on FST floating time was not observed (F_(4,81)_ = 2.184, *p* > 0.05) (see Supplementary Fig. S2). Body weight gain did not vary between PLF and PSF mice. Both groups had a significantly higher body weight gain after chronic paroxetine treatment compared to VEH mice (F_(2,42)_ = 29.51, *p* < 0.0001). Correlation between body weight gain and FST floating time was not significant (VEH: r = −0.02, *p* = 0.876; PLF: r = −0.32, *p* = 0.405; PSF: r = −0.15, *p* = 0.169) (see Supplementary Fig. S2).

### Chronic paroxetine treatment differentially affects brain and plasma purine and pyrimidine metabolism pathways between sub-groups

#### Brain

**Figure 2:**
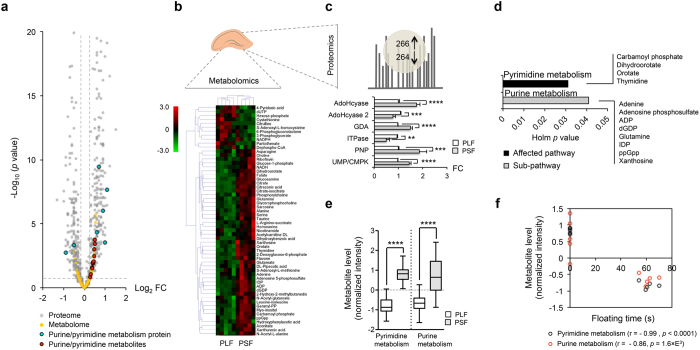
Hippocampal metabolite and protein expression alterations between PLF and PSF mice. (**a**) A volcano plot of hippocampal metabolome and proteome. Metabolites and proteins with log_2_|FC| > 0.3 and -log_10_(*p* value) > 1.3 were considered significant. Purine/pyrimidine metabolites and metabolism-related proteins were found to be significantly different between the sub-groups. (**b**) A heat map with combined profiles of SAM signatures (*q* < 0.05, FDR < 0.1) and their significant correlates (r > 0.7, *p* < 0.05). Heat map colors denote normalized metabolite intensity, *n* = 5/group. (**c**) Identification of purine and pyrimidine metabolism pathway proteins in proteomics analysis. Purine and pyrimidine metabolisms protein levels were significantly different between the PLF and PSF mice (*p* < 0.05), *n* = 3/group. (**d**) Metabolomics analysis identified purine and pyrimidine metabolisms as affected pathway and sub-pathway, respectively (Holm adjusted *p* < 0.05, FDR < 0.05). (**e**) Average level difference of purine and pyrimidine metabolites between PLF and PSF group was shown by box plots with whiskers min to max. (**f**) Average purine and pyrimidine pathways metabolite levels were strongly correlated with FST floating time. Data are expressed as the mean ± SEM. **p < 0.01, ***p < 0.001, ****p < 0.0001 (two-tailed *t*-test). Pearson correlation coefficients (r) with *p* values are indicated under the correlation graphs.

After clustering paroxetine-treated mice into long-floating and short-floating groups, we performed metabolomics and proteomics analyses of the hippocampus. Both -omics analyses showed purine/pyrimidine metabolites and proteins with significant differences between the two groups (log_2_|FC| > 0.3, -log_10_(*p* value) > 1.3) (Fig. 2a). In metabolomics analysis, significant analysis of microarrays (and metabolites) (SAM) and SAM-driven correlation analysis revealed that chronic paroxetine treatment differentially affected the hippocampal metabolome of the PLF and PSF mice (Fig. 2b). Sixteen metabolites were differentially regulated showing significantly higher levels in PSF compared to PLF mice (*q* < 0.05, FDR < 0.1). In addition, we found significant correlations with 42 other hippocampal metabolites (r > 0.7, FDR < 0.1) (see Supplementary Fig. S3). In proteomics analysis, we found that purine/pyrimidine metabolism proteins including S-adenosyl-L-homocysteine hydrolase (AdoHcyase), S-adenosyl-L-homocysteine hydrolase 2 (AdoHcyase 2), guanine deaminase (GDA), inosine triphosphate pyrophosphatase (ITPase), purine nucleoside phosphorylase (PNP) and UMP-CMP kinase (UMP/CMPK) were differentially expressed between the PLF and PSF groups (Fig. 2c).

The pyrimidine metabolism pathway was enriched with four metabolites from hippocampal SAM analysis (carbamoyl phosphate, dihydroorotate, orotate and thymidine). Eight correlates of SAM signatures (adenine, adenosine 5-phosphosulfate, ADP, dGDP, glutamine, IDP, ppGpp, xanthosine) enriched the purine metabolism pathway (Holm adjusted *p* < 0.05, FDR < 0.05) (Fig. 2d). Average levels of purine and pyrimidine metabolites were significantly higher in PSF than PLF mice and were strongly correlated with FST floating time (Fig. 2e,f). Purine and pyrimidine metabolite levels were higher in the PSF compared to PLF groups and with the exception of adenine and adenosine phosphosulfate showed a significant negative-correlation with FST floating time (see Supplementary Fig. S4). Metabolomics analysis of the prefrontal cortex did not result in any metabolite and pathway differences distinguishing the PLF and PSF groups.

#### Plasma

**Figure 3:**
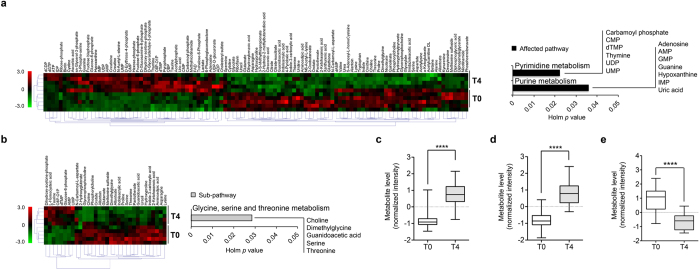
Chronic paroxetine treatment induced differential metabolome alterations in plasma. Heat maps and identified pathways of (**a**) PSF and (**b**) PLF groups comparing metabolome at baseline (T0) and following 28 days of treatment (T4). Purine and pyrimidine metabolisms were the only affected pathways in the PSF group. In the PLF group, correlates of SAM signatures identified glycine, serine and threonine metabolism as a sub-pathway (Holm adjusted *p* < 0.05, FDR < 0.05). Heat map colors denote normalized metabolite intensity, *n* = 5/group. Chronic paroxetine treatment induced average level changes of (**c**) purine and (**d**) pyrimidine pathways metabolites in the PSF group, and (**e**) glycine/serine/threonine pathway metabolites in PLF group between T0 and T4, *n* = 5/group. Metabolite levels are expressed with Box plots with whiskers min to max. ****p < 0.0001 (two-tailed *t*-test).

To delineate peripheral metabolome changes related to chronic paroxetine treatment response, plasma metabolite levels were investigated in the PLF and PSF groups at baseline (T0) and following 28 days of drug treatment (T4). While metabolomic profiles between PLF and PSF groups showed no significant differences both at T0 and T4, the PSF group exhibited profound differences with 43 significant metabolite level changes between T0 and T4 and another 71 metabolites that were strongly correlated (r > 0.7, FDR < 0.1) (see Supplementary Fig. S5). In the PSF group pyrimidine metabolism was enriched with 6 metabolites (carbamoyl phosphate, CMP, dTMP, thymine, UDP, UMP) and another 7 metabolites (adenosine, AMP, GMP, guanine, hypoxanthine, IMP, uric acid) enriched the purine metabolism pathway (Holm adjusted *p* < 0.05, FDR < 0.05) (Fig. 3a). The PLF group exhibited smaller metabolome changes compared to the PSF group. Only 4 metabolites were significantly altered after chronic paroxetine treatment according to SAM with another 32 metabolites highly correlated (r > 0.7, FDR < 0.1) (see Supplementary Fig. S5). While SAM signatures revealed no significantly affected pathways for the PLF group, glycine, serine and threonine metabolism was enriched for 5 correlates (choline, dimethylglycine, guanidoacetic acid, serine and threonine) (Holm adjusted *p* < 0.05, FDR < 0.05) (Fig. 3b). Plasma metabolite levels of identified pathways were significantly altered after mice had been treated chronically with paroxetine (Fig. 3c–e). Significant purine and pyrimidine metabolite level changes between T0 and T4 were observed only in the PSF mice (see Supplementary Fig. S6). Metabolite level changes of glycine, serine and threonine metabolism occurred both in PLF and PSF mice (see Supplementary Fig. S6). Plasma metabolite level changes did not show a significant correlation with FST floating time except for uric acid (see Supplementary Fig. S7). Seven plasma metabolites (2,3-dihydroxzbenzoic acid, aminoadipic acid, choline, pantothenate, taurine, threonine, uracil) were common and found to be regulated to a similar extent in both PLF and PSF groups (see Supplementary Fig. S8).

### Purine and pyrimidine metabolism pathways protein analysis

**Figure 4:**
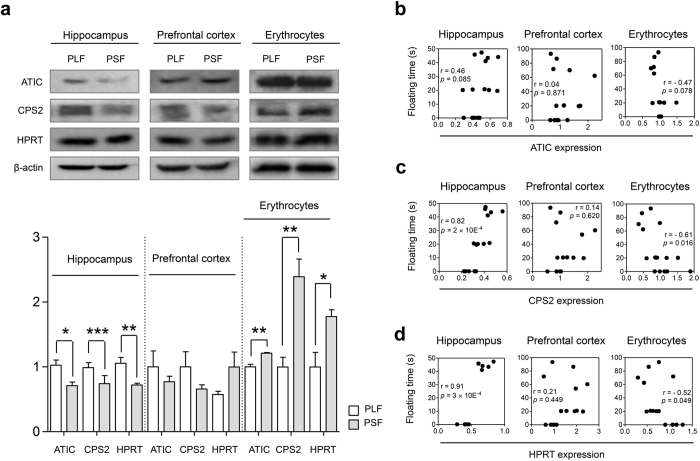
Chronic paroxetine treatment induces differential alteration of ATIC, CPS2 and HPRT protein expression in mice. (**a**) Western blot and densitometry analyses of CPS2 and HPRT protein levels in the hippocampus, prefrontal cortex and erythrocytes. The image represents cropped blots showing the relevant protein bands. All gels and blots were run under the same experimental conditions. Cropping lines are indicated in full-length blots of Supplementary Fig. S9. Hippocampal and erythrocytic ATIC, CPS2 and HPRT proteins showed significant expression level differences between the PLF and PSF groups, *n* = 5/group. (**b**) ATIC protein level showed moderate correlation with FST floating time. Correlation of (**c**) CPS2 and (**d**) HPRT protein levels with FST floating time was significant in the hippocampus and erythrocytes, *n* = 15. The cropped blots were used for the Fig. 4a. The gels have been run under the same experimental conditions. Protein expression levels were normalized with β-actin. Data are expressed as the mean ± SEM. *p < 0.05, **p < 0.01, ***p < 0.001 (two-tailed *t*-test). Pearson correlation coefficients (r) with *p* values are indicated in the correlation graphs.

Aminoimidazole-4-carboxamide ribonucleotide transformylase/IMP cyclohydrolase (ATIC), carbamoyl phosphate synthase 2 (CPS2) and hypoxanthine-guanine phosphoribosyltransferase (HPRT) protein expression in the hippocampus, prefrontal cortex and erythrocytes were assessed to validate differentially affected pathways between the PLF and PSF groups (Fig. 4a, see Supplementary Fig. S9). In the hippocampus, ATIC, CPS2 and HPRT protein levels showed significant differences between groups. Compared to the PLF group the PSF group had significantly reduced ATIC, CPS2 and HPRT protein expression levels (ATIC: t = 3.304, df = 8, *p* < 0.05, CPS2: t = 1.702, df = 8, *p* < 0.001, HPRT: t = 3.488, df = 8, *p* < 0.01). In the prefrontal cortex protein expression showed no difference between the two groups. For validation of peripheral pathways identified in plasma, erythrocytes were chosen as source. Interestingly, erythrocytic ATIC, CPS2 and HPRT proteins were also differentially expressed between the PLF and PSF groups. Erythrocytic ATIC and CPS2 protein expression in the PSF mice was 1.2-fold and 2.4-fold higher than in the PLF mice, respectively (ATIC: t = 4.991, df = 8, *p* < 0.01, CPS2: t = 4.484, df = 8, *p* < 0.01). HPRT protein levels were 1.7-fold higher in the PSF compared to the PLF group (t = 3.145, df = 8, *p* < 0.05). Furthermore, FST floating time significantly correlated with CPS2 and HPRT protein expression levels in the hippocampus and erythrocytes, but not in the prefrontal cortex. Correlation between FST floating time and ATIC protein levels also showed strong tendency in the hippocampus and erythrocytes (Fig. 4b–d).

### Analysis of peripheral patient specimens

Next, we sought to corroborate our findings on pyrimidine and purine metabolism in patients chronically treated with antidepressants. For this purpose we carried out experiments with PBMCs isolated from patients.

**Figure 5:**
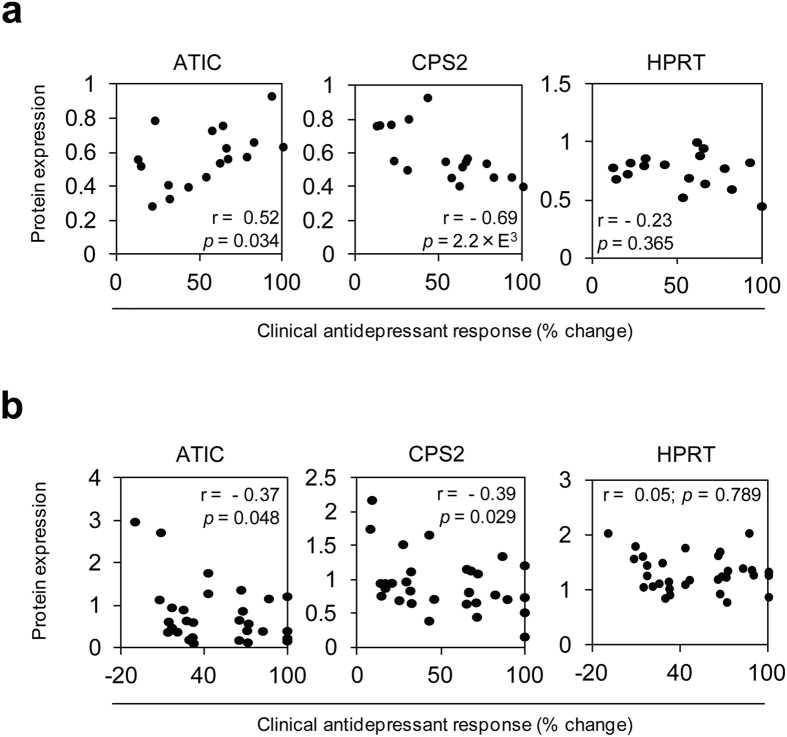
Correlation of ATIC, CPS2 and HPRT protein levels with clinical antidepressant treatment response. (**a**) Depression patients’ PBMCs collected after 4–6 weeks of antidepressant treatment were analyzed for ATIC, CPS2 and HPRT protein expression. ATIC and CPS2 protein levels significantly correlated with clinical antidepressant response, *n* = 17. (**b**) PBMCs from inpatients with depression were collected at admission. Cells were *ex vivo* cultivated and treated with paroxetine for 2 days. After treatment, ATIC and CPS2 protein levels significantly correlated with clinical antidepressant response, *n* = 32. Pearson correlation coefficients (r) with *p* values are indicated in the correlation graphs.

First, the same pyrimidine and purine pathway proteins analyzed in mice, ATIC, CPS2 and HRPT, were assessed in PBMCs obtained from antidepressant responder and non-responder patients of the MARS study. In PBMCs collected after 4–6 weeks of antidepressant treatment, ATIC and CPS2 protein levels were significantly correlated with clinical antidepressant treatment response (ATIC: r = 0.52, *p* = 0.034; CPS2: r = −0.69, *p* = 0.002; HPRT: r = −0.23, *p* = 0.365) (Fig. 5a).

To further investigate pharmacological effects of paroxetine we also performed *ex vivo* experiments with patients’ PBMCs. Cells collected from patients upon admittance were cultivated and treated with paroxetine for 2 days. As had been the case for the *in vivo* PBMCs analysis, ATIC and CPS2 protein levels also significantly correlated with patients’ clinical antidepressant response when their cultured PBMCs were treated with paroxetine (ATIC: r = −0.37, *p* = 0.048; CPS2: r = −0.39, *p* = 0.029; HPRT: r = 0.05, *p* = 0.789) (Fig. 5b).

## Discussion

In the present study we observed a heterogeneous pharmacological treatment response towards paroxetine in an inbred mouse strain. Using FST floating time, an indicative parameter for antidepressant-like activity^7^,^8^,^9^, we were able to stratify mouse sub-groups that differed in their response following chronic paroxetine administration. We submit that paroxetine-treated mice exhibiting no FST floating time difference with vehicle-treated mice are non-responsive towards SSRI treatment. We then examined metabolite and protein profiles in short-floating and long-floating mouse sub-groups representing paroxetine responders and non-responders, respectively, aiming to delineate molecular pathways associated with the SSRI response.

To examine the relevance of covariates drug levels, age and body weight gain were assessed. Paroxetine concentrations in whole brain and plasma were analyzed to check for a possible association of drug levels with antidepressant-like activity. Previous studies have reported a significant relationship between plasma levels and therapeutic response towards paroxetine^10^. In our study we did not find any paroxetine concentration differences between PLF and PSF mice in either whole brain or plasma and drug levels did not correlate with FST floating time. This suggests that paroxetine levels in whole brain and plasma are irrelevant for the observed differential drug treatment response in our long-term treatment setting. We also considered animal age and its relationship with paroxetine response. Age-dependent outcome of SSRI treatment has been assessed with regard to adverse effects of drug treatment by comparing antidepressant-induced behavioral response of juvenile or adolescent and adult rodents^11^,^12^. In the current study all mice reached adulthood prior to being subjected to experiments (>8 weeks). Although significant FST floating time differences were observed for mice between 18 and 20 weeks of age, no general age effect on FST floating time was detected. The relationship between body weight gain and chronic paroxetine treatment response was also investigated. Chronic paroxetine treatment induced a significant increase of body weight. However, body weight gain and drug treatment response for each group did not correlate significantly.

To further characterize the PLF and PSF sub-groups we assessed female urine sniffing time, a behavioral parameter pertinent to evaluate SSRI treatment effect in mice^13^,^14^. While the PLF and PSF groups did not show any difference of female urine sniffing time prior to being treated with paroxetine, chronic paroxetine treatment induced a differential behavioral effect between the groups. This finding indicates that the different behavior between mice is not inherent, but is the result of chronic paroxetine treatment.

We performed metabolomics and proteomics analyses of the hippocampus to profile brain metabolite and protein differences between PLF and PSF mice.

In metabolomics analysis, significant metabolites enriched the pyrimidine metabolism as a differentially affected pathway between the two groups. Significant correlates of SAM signatures were investigated and revealed purine metabolism as an interacting sub-pathway. In proteomics analysis, purine/pyrimidine metabolism-related proteins were quantified with significant differences between the groups. Integrated metabolomics and proteomics data implicate a differential effect of chronic paroxetine treatment on mouse hippocampal purine and pyrimidine metabolism.

Purine and pyrimidine metabolites and their receptors have previously been shown to be associated with various neuropsychiatric disorders. The anti-purinergic drug suramin was found to reverse autism-like behaviors and metabolism in mice^15^. Polymorphisms of the P2RX7 gene, which encodes a purinergic ion channel, have been associated with the development of MDD^16^ In addition, low brain purine levels were found in female depressed patients responding to treatment with the SSRI fluoxetine^17^. Uridine, a pyrimidine metabolite, has been shown to have antidepressant-like activities in mice^18^.

Hippocampal metabolome profiling also implicated other metabolites with elevated levels in PSF mice that have antidepressant-like activity. Folate has been shown to have an antidepressant-like effect in mice^19^ and low folate levels were found to be associated with MDD^20^ L-Methylfolate, the active metabolite of folate is used for patients with MDD who partially respond or do not respond to SSRIs^21^. Myo-inositol has been identified as a potential biomarker of SSRI treatment response and innate anxiety disorder^22^,^23^,^24^ and has been shown to have anxiolytic and antidepressant-like effects in both animals and humans^25^,^26^. Flavones are also known to have an antidepressant-like effect^27^,^28^. The elevated levels of folate, myo-inositol and flavones that we found in the hippocampus of PSF mice might be of relevance for the favorable paroxetine response.

Peripheral specimens such as plasma and blood cells are the preferred biomarker sources for assessing the antidepressant treatment response in the clinical laboratory^29^,^30^,^31^. We compared the plasma metabolome of the two mouse sub-groups, PLF and PSF, at baseline and after 28 days of treatment (T0 and T4) with the aim of identifying differentially affected pathways and potential biomarker candidates in the periphery. Plasma metabolome changes over time resulted in group-specific profiles. Major metabolite level alterations and elevation of purine and pyrimidine metabolites were observed in the PSF group. In contrast, the plasma metabolome was minimally affected by chronic paroxetine treatment in the PLF group. Despite the fact that the glycine, serine and threonine metabolism pathway was specifically enriched as a sub-pathway of PLF group, significant metabolite level changes were observed in both sub-groups. We also found plasma metabolite biosignatures commonly regulated between the PLF and PSF groups. In particular levels of choline and threonine which are part of the glycine, serine and threonine metabolism pathway were changed both in the PLF and PSF groups after chronic paroxetine treatment. Thus, metabolites of glycine, serine and threonine metabolism do not seem to serve as a useful biosignature for the chronic paroxetine treatment response.

In contrast to hippocampal purine/pyrimidine metabolites, plasma metabolite level changes were not correlated with FST floating time. Small plasma metabolite level differences between the PLF and PSF groups might be responsible for non-significant correlation. Despite non-significant correlation between plasma metabolite level changes and chronic paroxetine treatment response, group-specific analysis showed PSF-group specific pathway enrichment and significant metabolite changes.

In previous studies our -omics analyses revealed that energy metabolism-related pathways were significantly altered by chronic paroxetine- compared to vehicle-treated mice^22^,^29^. The current -omics analyses were performed to compare PLF and PSF mouse groups and identified purine and pyrimidine metabolisms as the main distinguishing pathways. However, ATP/ADP and NAD^+^/NADH ratios in the current study indicate that energy metabolism might also distinguish the PLF and PSF groups (see Supplementary Fig. S10).

Based on our integrated -omics data that implicate purine and pyrimidine metabolism pathways to be involved in paroxetine response we next wanted to corroborate these findings through the analysis of proteins that are part of these pathways. Based on the observed differences of carbamoyl phosphate and IMP levels between the PLF and PSF groups, ATIC, CPS2 and HPRT proteins were analyzed. CPS2 catalyzes early steps of carbamoyl phosphate synthesis in the pyrimidine biosynthesis pathway. ATIC and HPRT play a central role in synthesis and conversion of inosine monophosphate (IMP), the end product of the purine biosynthesis pathway. For pathway validation we chose hippocampus, prefrontal cortex and erythrocytes and compared ATIC, CPS2 and HPRT protein levels between the PLF and PSF groups. Western blot analyses revealed that hippocampal and erythrocytic ATIC, CPS2 and HPRT proteins were differentially expressed between the two groups while prefrontal cortex protein expression showed no difference. Hippocampal and erythrocytic ATIC, CPS2 and HPRT protein levels were also highly correlated with FST floating time while no significant correlation was observed in the prefrontal cortex. This indicates that different purine and pyrimidine metabolism pathway activities between PLF and PSF groups might be specific for the hippocampus.

Interestingly, we observed an inverse relationship between hippocampal and erythrocytic protein expression for the three proteins. Inconsistent biosignature expression patterns in brain and peripheral tissues have been found in other cases related to psychiatric disorders. Brain and blood BDNF levels were inversely correlated in a genetic rat model of depression^32^. Brain and white blood cell p11 protein levels also showed an inverse relationship^33^. Inverse myo-inositol levels between hippocampus and plasma were also reported previously in mice chronically treated with paroxetine^22^. In the current study we found an inverse correlation trend for carbamoyl phosphate and IMP levels between hippocampus and plasma. This might be caused by the observed inverse relationship of ATIC, CPS2 and HPRT enzyme expression levels between hippocampus and erythrocytes (see Supplementary Fig. S11).

To address the question whether the pathways for antidepressant response identified in the mouse are also relevant for patients’ response, PBMCs from antidepressant responder and non-responder patients were analyzed for ATIC, CPS2 and HPRT protein expressions. PBMCs collected after 4–6 weeks of antidepressant treatment showed significant correlation between ATIC and CPS2 protein expression levels and HDRS score change between baseline and following chronic antidepressant treatment. Whereas PBMCs ATIC protein expression showed a similar pattern as the one observed in mouse erythrocytes, PBMCs CPS2 protein expression resembled that of mouse hippocampus.

In *ex vivo* experiments, PBMCs ATIC protein levels were negatively correlated with clinical antidepressant response. The observed discrepancy between *in vivo* and *ex vivo* PBMCs ATIC protein expression could be due to different treatment conditions (chronic vs. subchronic) and exposure to multiple types of drugs. PBMCs’ CPS2 protein expression consistently showed negative correlation with mouse hippocampal protein expression both in *in vivo* and *ex vivo*. ATIC and CPS2 proteins may thus represent candidate biomarkers to predict clinical antidepressant treatment response. For HPRT protein, we failed to determine a significant correlation between clinical antidepressant response and PBMCs protein levels both *in vivo and ex vivo*.

Based on evidence from the literature microRNAs (miRNAs) may be relevant for the distinct regulation of purine/pyrimidine metabolisms in the PLF and PSF mouse groups. Feng *et al*. have shown that miR-1 and miR-133a-3p regulate purine and pyrimidine metabolic pathways^34^ and the purine metabolism gene *GART* is regulated by 16 miRNAs^35^. Since SSRIs impact miRNA levels^36^ this may explain the observed differences in the purine and pyrimidine metabolisms upon chronic paroxetine treatment.

The current study used wild-type stress naïve DBA/2J mice to investigate the pharmacological heterogeneity of the antidepressant response. Wild-type stress naïve rodents have been used previously to evaluate antidepressant-like effects^37^,^38^,^39^. An extension of our studies using an animal model with a depression-like phenotype would further validate the identified pathways affected by the antidepressant treatment response and add relevant information for the antidepressant treatment of patients.

What roles purine and pyrimidine metabolic pathways play in the underlying mechanism of SSRI response requires further studies with compounds able to modulate pathway activity. Pathway modulators may lead to novel drugs with antidepressant activities. In addition, purine and pyrimidine metabolic pathway activities could be used as a biosignature for the antidepressant treatment response. With biomarkers for antidepressant treatment response patient sub-groups can be stratified and this will render clinical decision more objective. Whether the identified pathways can lead to predictive biosignatures for antidepressant treatment response remains to be investigated.

## Methods

### Animal Housing and Husbandry

The experiments were carried out with male DBA/2J mice (Charles River Laboratories, Chatillon-sur-Chalaronne, France). All animals were between 8–10 weeks old and single housed for at least one week prior to the beginning of the experiments. Mice were held under normal light and temperature conditions (12 light: 12 dark light cycle, lights on at 7 pm, temperature at 23 ± 2 °C, and humidity at 55 ± 5%) with standard bedding and nesting material, in polycarbonate cages (21 × 15 × 14 cm). Water and Altromin 1324 standard mouse chow (Altromin GmbH, Lage, Germany) were provided *ad libitum*. All procedures were carried out in accordance with the European Communities Council Directive 2010/63/EU and approved by the committee for the Care and Use of Laboratory animals of the Government of Upper Bavaria, Germany.

### Drug Administration

In preliminary studies we investigated the minimum effective dosage of paroxetine in DBA/2J mice to minimize drug-induced side effects. Two doses of paroxetine pills (either 2 × 1 mg/kg/day or 2 × 5 mg/kg/day) were given to mice for 28 days. In forced swim test (FST), only higher dose (2 × 5 mg/kg/day) of paroxetine treatment induced a significant floating time increase. Therefore, mice were treated with vehicle or 5 mg/kg paroxetine pills (Paroxetine hydrochloride; Carbone Scientific, London, UK) for 28 days twice a day. Animals were randomly distributed to the vehicle or paroxetine treated experimental groups. Vehicle or paroxetine was voluntarily self-administered via customized palatable pellets (40 mg PQPellets, Phenoquest AG, Martinsried, Germany). Animals that did not take the treatment properly were excluded from further analysis.

### Mouse brain and blood collection

Blood was collected at least one month before commencing paroxetine treatment from retro-orbital puncture and after 28 days of paroxetine treatment through cardiac puncture or trunk blood. On day 29, the animals were subjected to a FST and sacrificed. Trunk blood and brains of the animals were collected and stored at −80 °C until further use. Blood was centrifuged to separate plasma and erythrocytes (1300 g, 10 min, 4 °C) before storage.

### Behavioral Analyses

#### Forced Swim Test

Mice were placed into a glass beaker (height 24 cm, diameter 13 cm) filled with water (21 ± 1 °C) up to a height of 15 cm, so that the animals were unable to reach the ground or escape for 6 min testing. Main parameter of interest is floating time scored by an experienced observer blind to treatment.

#### Female urine sniffing test

Mice were habituated to a cotton swab inserted into their home cage for 1 h prior to testing. Mice were exposed to a sterile cotton swab dipped into water for 3 min and after a 45 min inter trial interval. Then they were exposed to a sterile cotton swab dipped in estrous female urine from the same strain. Total sniffing time was recorded.

### Hierarchical Clustering Analysis (HCA)

HCA is a method to build and split different cluster hierarchies. It has been applied to identify sub-groups of cells and animals based on marker protein expression or behavioral parameters^40^,^41^. HCA was carried out with SPSS (SPSS version 21, IBM SPSS Inc., Chicago, IL, USA) to separate paroxetine-treated sub-groups of mice based on FST floating time. Animals with extreme FST floating times were selected from the PLF and PSF groups for -omics analyses. Proteomics and metabolomics profiles from individual animals were used with no data pooling.

### Paroxetine Measurements

Mouse whole brains were homogenized in a fivefold volume of phosphate buffered saline (PBS) containing “Complete Protease Inhibitor Cocktail Tablets” (Roche, Penzberg, Germany) using a Dispomix Drive (Medic Tools AG, Zug, Switzerland). All samples were prepared using Ostro protein precipitation and phospholipid removal plates (Waters, Eschborn, Germany). Plasma and brain homogenates were analyzed by LC-MSMS using an Agilent 1100 Series (Agilent, Waldbronn, Germany) liquid chromatograph interfaced with an Applied Biosystems API 4000 (ABSciex, Darmstadt, Germany) triple quadrupole mass spectrometer. Deuterated paroxetine (Paro-D6) was used as internal standard. Five μl samples were loaded and gradient eluted from an Accucore RP-MS 2.6 μm column (2.1 × 50 mm, Thermo Scientific, Dreieich, Germany) at a flow rate of 0.3 ml/min and 30 °C (eluent A: methanol, 10 mM ammonium formate, 0.1% formic acid; eluent B: 10 mM ammonium formate, 0.1% formic acid). Gradient: 0–0.5 min 20% A, 0.5–2 min 20–90% A, 1 min held at 90% A, 3–3.5 min 90–20% A and 3.5–8 min 20% A. The ion source was operated in positive mode at 500 °C and multiple reaction monitoring (MRM) collision-induced dissociation (CID) was performed using nitrogen collision gas. The collision energy was set to 29 V for paroxetine and 33 V for Paro-D6. The transitions monitored during analysis were *m/z* 330 → 192 for paroxetine and *m/z* 336 → 198 for Paro-D6.

### Proteomics Analysis

Mouse hippocampus was homogenized in a buffer containing 2M NaCl, 10 mM HEPES/NaOH, 1 mM EDTA and protease inhibitor cocktail tablets (Roche Diagnostics, Mannheim, Germany) and phosphatase inhibitors (Sigma, St. Louis, MO, USA). Homogenates were sonicated with an ultra-sonicator (Branson, Danbury, CT, USA) and centrifuged (16100 g, 20 min, 4 °C). The protein concentration was determined by Bradford assay.

Protein extracts were mixed with equal amounts of ^15^N-labeled DBA/2 mouse hippocampal protein extract^24^. Fourty μg of ^14^N- ^15^N hippocampal protein mixture was separated in a 10% SDS-PAGE gel and stained with Coomassie Brilliant Blue R-250 (BioRad, Hercules, CA, USA) overnight. After destaining and cutting the gel lane into slices, tryptic peptides were produced and extracted as previously described^42^. Extracted peptides were analyzed by liquid chromatography-electrospray tandem mass spectrometry (LC-MS/MS) using a nanoflow HPLC-2D system (Eksigent, Dublin, California) coupled online to an LTQ-Orbitrap mass spectrometer (Thermo Fisher Scientific, Bremen, Germany). Protein identification and quantitation were performed as described previously^22^.

### Metabolomics Analysis

Mouse brain and plasma metabolites were extracted and analyzed with targeted metabolomics as previously described^43^. Animals from the same cohort were used for all metabolomics analyses.

### Paroxetine treatment of patient peripheral blood mononuclear cells

Blood of patients with major depression disorder was collected between 08:00 and 09:00 h within 5 days after admittance. PBMCs were prepared as described previously^44^. Cells were treated with 120 ng/ml paroxetine for 2 days according to the consensus guidelines for therapeutic drug monitoring in psychiatry^45^.

### Protein level quantitation in cultivated PBMCs

In *ex vivo* cultivated PBMCs, ATIC, CPS2, HPRT and β-actin protein levels were detected and quantitated with an automated capillary immunoassay system, Simple Western^TM^ (ProteinSimple, Santa Clara, CA, USA). PBMCs lysates were prepared according to manufacturer’s instruction. All following steps were fully automated. Protein quantitation data were normalized with β-actin.

### Western Blot Analysis

Mouse hippocampus, prefrontal cortex and erythrocytes were homogenized with the same buffer used for proteomics sample preparation, or RIPA buffer containing protease inhibitor cocktail tablets (Roche Diagnostics, Mannheim, Germany) and phosphatase inhibitors (Sigma, St. Louis, MO, USA). Patient PBMCs were homogenized with RIPA buffer containing protease and phosphatase inhibitors. Homogenates were sonicated and centrifuged (16100 g, 20 min, 4 °C). Bradford assay was used to quantify extracted protein concentration. Proteins were separated in 10–15% gradient SDS-PAGE gels. Subsequently, they were transferred to a PVDF membrane (Millipore, Billerica, MA, USA). After blotting, the membrane was blocked with 5% skim milk solution for 1 h at room temperature and incubated with either anti-aminoimidazole-4-carboxamide ribonucleotide transformylase/IMP cyclohydrolase (ATIC) antibody (1:500, Santa Cruz, Dallas, TX, USA) or anti-carbamoyl phosphate synthase 2 (CPS2) antibody (1:500, Santa Cruz, Dallas, TX, USA) or anti-hypoxanthine-guanine phosphoribosyltransferase (HPRT) antibody (1:500, Sigma, St. Louis, MO, USA) or β-actin antibody (1:4000, Sigma, St. Louis, MO, USA) at 4 °C overnight. The membranes were washed and then incubated with horseradish peroxidase (HRP) conjugated-secondary antibodies. The blots were developed with Luminata^TM^ Forte Western HRP Substrate (Millipore, Billerica, MA, USA). Images were acquired by ChemiDoc^TM^ MP imaging system (Bio-Rad Laboratories, Munich, Germany). Densitometric data analyses were carried out with ImageJ software (National Institute of Health, USA).

### Statistical Analysis

Statistical analysis of behavioral data in FST/FUST and covariates were performed with GraphPad Prism 5 (GraphPad Software, Inc., La Jolla, CA, USA). Student *t*-test, one-way or two-way ANOVA were used to assess statistical significance between groups. Pearson correlation coefficients (r) with *p* values were used to evaluate correlation between floating time and body weight gain. For the identification of significantly altered metabolites, metabolite peak intensities were median and auto-scaled normalized. Metabolites with missing values, 30 for the hippocampus and 27 for the prefrontal cortex in all replicates, were excluded from data analysis. Significant analysis of microarrays (and metabolites) (SAM) method was used to identify significantly altered metabolites (*q* < 0.05, FDR < 0.1).

Significantly altered metabolites were subjected to pathway enrichment analysis of MetaboAnalyst ( http://www.metaboanalyst.ca) to identify differentially affected pathways between the PLF and PSF groups. Pathways with Holm adjusted *p* < 0.05 and FDR < 0.05 were considered significantly affected. To identify sub-pathways interacting with a differentially affected pathway between the PLF and PSF groups, correlates of each SAM signature were combined and used to enrich relevant metabolic pathways using MetaboAnalyst (Pearson correlation coefficients (*r*) > 0.7, FDR < 0.1). Pathways with Holm adjusted *p* < 0.05 and FDR < 0.05 were considered significant. CPS2, HPRT and β-actin Western blot data were also analyzed with GraphPad Prism 5. Two-tailed *t*-test was used to evaluate the difference between the PLF and PSF groups. Data were expressed as the mean ± the standard error of the mean (SEM). Statistical data were considered significant at *p* < 0.05. D’Agostino & Pearson omnibus normality test was used to check normal distribution of the data.

### Patient samples

For *in vivo* and *ex vivo* studies, two distinct PBMC batches from different individuals were chosen. Peripheral blood mononuclear cells (PBMCs) obtained from 17 participants of the Munich Antidepressant Response Signature (MARS) study were included for assessing protein expression levels (see Supplementary Table S1). PBMCs from 32 individuals were subjected to *ex vivo* cultivation and paroxetine treatment (see Supplementary Table S2). Diagnosis was conducted according to DSM-IV criteria, and all participants were diagnosed as having MDD (see Supplementary Table S3). Depression severity was evaluated using the 21-item Hamilton Depression Rating Scale (HDRS). Responder and non-responder patients were classified based on clinical antidepressant treatment response corresponding to minimal 50% reduction in HDRS score between baseline (T0) and after 6 weeks of admission (T6). The Munich Antidepressant Response Signature (MARS) study was approved by the local ethics committee of the Ludwig-Maximilians-University Munich, Germany (approval no. 318/00), and the study was carried out in accordance with the principles of Good Clinical Practice and with the latest edition of the Declaration of Helsinki. All patients provided written informed consent before study inclusion.

## Additional Information

**How to cite this article**: Park, D. I. *et al*. Purine and pyrimidine metabolism: Convergent evidence on chronic antidepressant treatment response in mice and humans. *Sci. Rep.* **6**, 35317; doi: 10.1038/srep35317 (2016).

## Supplementary Material

Supplementary Information

Supplementary Table 3
