# Supplementary material for: Purine and pyrimidine metabolism: Convergent evidence on chronic antidepressant treatment response in mice and humans
Source: Sci Rep. 2016 Oct 12;6:35317. doi: 10.1038/srep35317 (PMC5059694; doi:10.1038/srep35317)
Supplement: Supplementary Information [file srep35317-s1.pdf]

Supplementary Information

Title: Purine and pyrimidine metabolism: Convergent evidence on chronic antidepressant treatment response in mice and humans

Dong Ik Park[1,7], Carine Dournes[2,7], Inge Sillaber[3], Manfred Uhr[4], John M. Asara[5], Nils C. Gassen[2], Theo Rein[2], Marcus Ising[4], Christian Webhofer[1], Michaela D. Filiou[2], Marianne B. Müller[1,6], Christoph W. Turck[1,*]

[1] Max Planck Institute of Psychiatry, Department of Translational Research in Psychiatry, 80804, Munich, Germany; [2] Max Planck Institute of Psychiatry, Department of Stress Neurobiology and Neurogenetics, 80804 Munich, Germany; [3] Phenoquest AG, 82152 Martinsried, Germany; [4] Max Planck Institute of Psychiatry, Department of Clinical Research, 80804 Munich, Germany; [5] Division of Signal Transduction, Beth Israel Deaconess Medical Center, and Department of Medicine, Harvard Medical School, Boston, MA 02115; [6] Experimental Psychiatry, Department of Psychiatry and Psychotherapy & Focus Program Translational Neuroscience, Johannes Gutenberg University Medical Center, 55128 Mainz, Germany

[7] These authors contributed equally to this work.

Supplementary Results

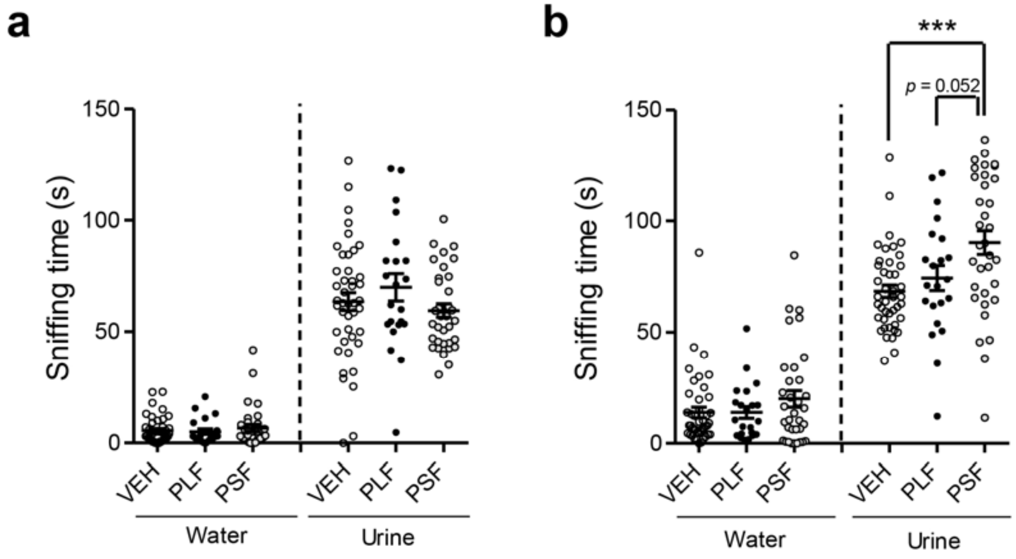

Supplementary Figure S1. The effect of chronic paroxetine treatment on female urine sniffing test (FUST). Sniffing time at (**a**) baseline and (**b**) after 28 days of paroxetine treatment. Chronic paroxetine treatment induced slightly significant difference of sniffing time between paroxetine-treated long- time floating (PLF) and paroxetine-treated short-time floating (PSF) groups. *n(VEH/PLF/PSF)* = 43/22/34. *p < 0.05 (two-tailed *t*-test), ***p < 0.001 (one-way ANOVA with Tukey's test for multiple comparisons).

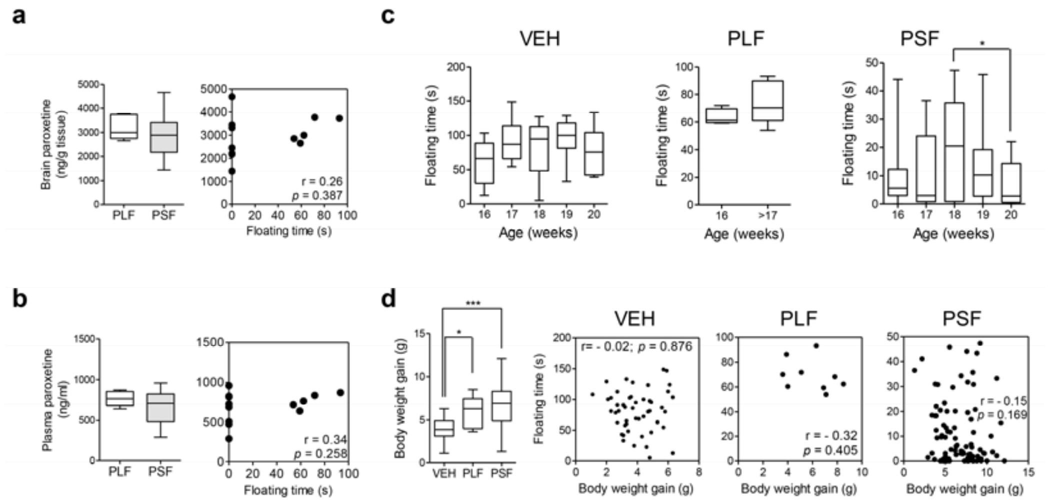

Supplementary Figure S2. Covariates analysis. Paroxetine levels in (**a**) whole brain and (**b**) plasma. PLF and PSF groups did not show significant paroxetine levels differences, *n*(PLF/PSF)=5/8. The effect of (**c**) age and (**d**) body weight gain on FST floating time. Significant interactions of age and body weight gain with floating time were not observed. *n*(VEH/PLF/PSF)=50/9/86. Data are expressed as the mean ± SEM. *$p < 0.05$, ***$p < 0.001$ (one-way ANOVA with Tukey's test for multiple comparisons). Pearson correlation coefficients (r) with P values are indicated in the correlation graphs.

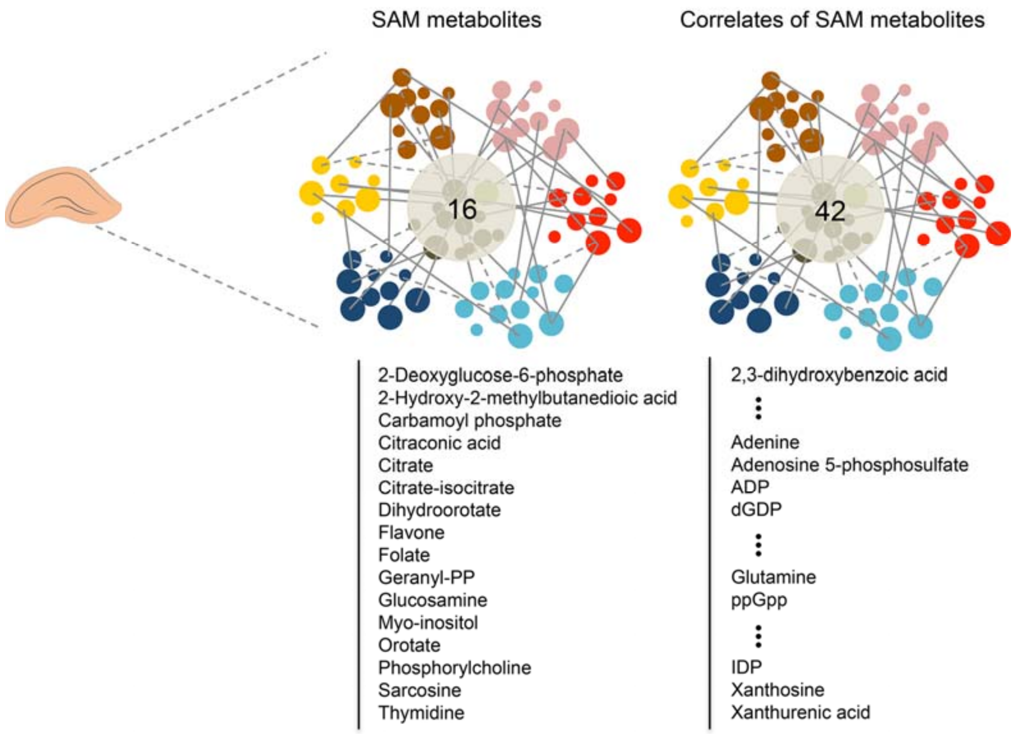

Supplementary Figure S3. Hippocampal SAM metabolites (q < 0.05, FDR < 0.1) and their significant correlates (r > 0.7, $p < 0.05$).

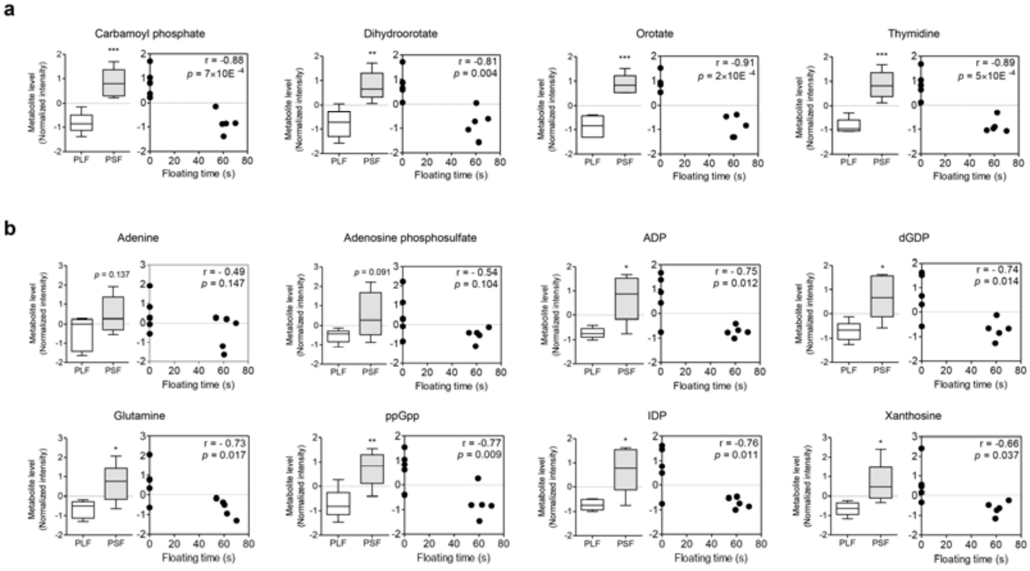

Supplementary Figure S4. Levels of hippocampal metabolites that are part of (**a**) pyrimidine and (**b**) purine metabolism pathways and correlation with forced swim test (FST) floating time. n = 5/group. Bars represent mean ± SEM. *$p < 0.05$, **$p < 0.01$, ***$p < 0.001$ vs PLF (two-tailed t-test). Pearson correlation coefficients (r) with p values are indicated in the correlation graphs.

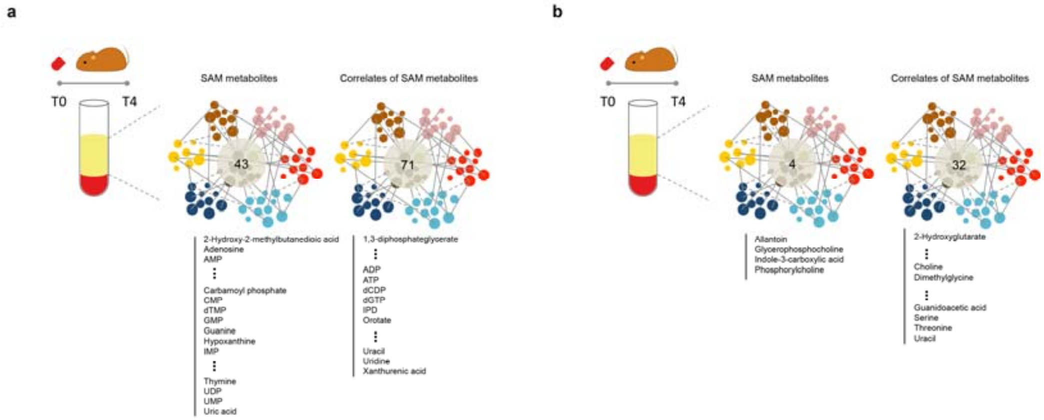

Supplementary Figure S5. Significant plasma metabolite level changes after chronic paroxetine treatment. Plasma SAM metabolites (q < 0.05, FDR < 0.1) and their significant correlates (r > 0.7, *p* < 0.05) in (a) PSF and (b) PLF groups.

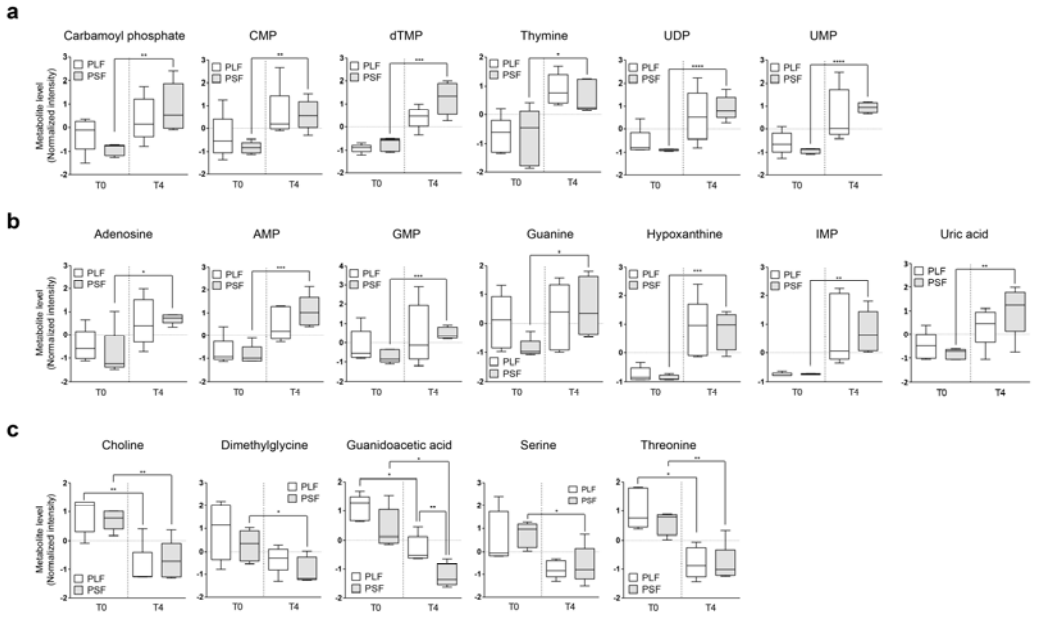

Supplementary Figure S6. Plasma pathway metabolites. (**a**) Pyrimidine and (**b**) purine metabolism metabolite levels in the PSF group were elevated by chronic paroxetine treatment. The metabolite levels in the PLF group did not show alterations after chronic paroxetine administration. (**c**) Both PLF and PSF mice showed that glycine, serine and threonine metabolite levels were significantly down-regulated by chronic paroxetine treatment. The PLF and PSF groups exhibited similar metabolite levels both at T0 and T4. n = 5/group. *p < 0.05, **p < 0.01, ***p < 0.001, ****p < 0.0001 (two-tailed t-test).

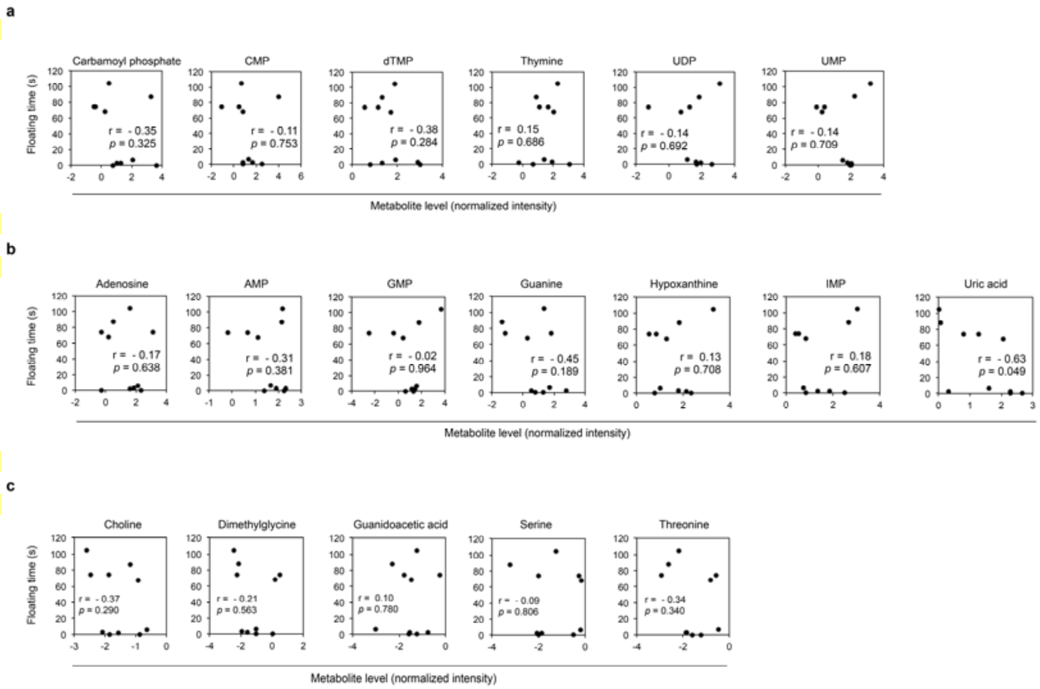

Supplementary Figure S7. Correlation of plasma pathway metabolite levels with FST floating time. Pearson correlation coefficients (r) with *p* values are indicated in the correlation graphs.

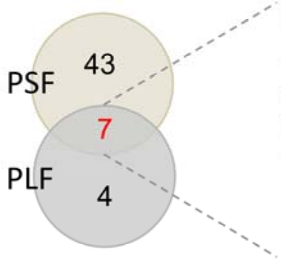

Metabolite	PLF		PSF	
	q value	FC	*q* value	FC
2,3-Dihydroxybenzoic acid	5.19E-02	0.55	2.04E-02	0.61
Aminoadipic acid	5.19E-02	0.58	3.27E-02	0.63
Choline	4.48E-02	0.54	2.40E-02	0.62
Pantothenate	4.83E-02	0.60	3.14E-02	0.60
Taurine	2.29E-02	1.94	2.21E-02	1.69
Threonine	4.42E-02	0.55	2.40E-02	0.61
Uracil	1.97E-02	0.50	1.69E-02	0.58

Supplementary Figure S8. Common plasma SAM signatures between PLF and PSF groups. q value < 0.05 in either PLF or PSF groups, FDR < 0.1.

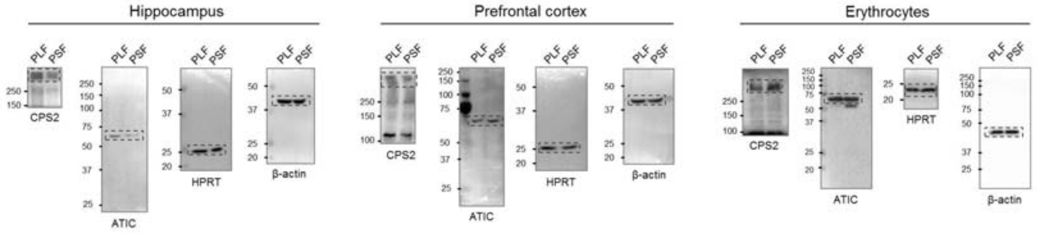

Supplementary Figure S9. Full-length western blot images of Figure 4a.

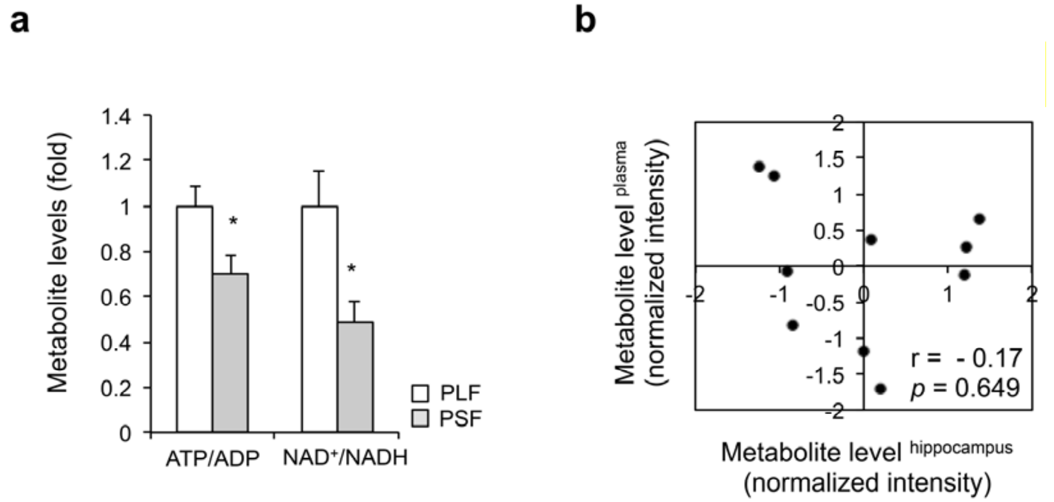

Supplementary Figure S10. Energy-related metabolite ratios and myo-inositol correlation between hippocampus and plasma. (**a**) ATP/ADP and NAD+/NADH ratios in the hippocampus between the PLF and PSF group. *n* = 5/group. (**b**) Myo-inositol correlation between hippocampus and plasma. *n* = 10. Bars represent mean ± SEM. *$p < 0.05$ (two-tailed *t*-test). Pearson correlation coefficients (r) with *P* values are indicated in the correlation graphs.

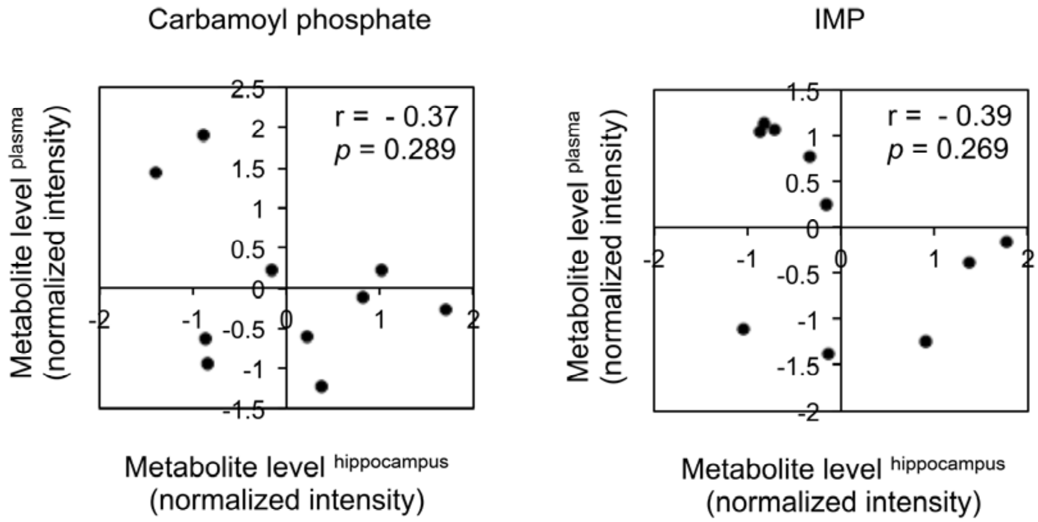

Supplementary Figure S11. Correlation between hippocampus and plasma metabolite levels. Pearson correlation coefficients (r) with *P* values are indicated in the correlation graphs.

Characteristic	Responders<br>Mean (± SD)	Non-responders<br>Mean (± SD)	Statistics
Age	49 (14)	49 (11.6)	p = 0.58
Males/Females	6/4	3/4	χ2 = 0.4857, p = 0.49
HDRS at admittance	23.8 (5.7)	22.5 (4.2)	p = 0.35
HDRS at T6	7 (3.6)	17.1 (4.2)	$p = 2.0 \times E^{4}$

HDRS, Hamilton Depression Rating Score
T6, after six weeks of admission

Supplementary Table S1. Demographic and clinical characteristics of antidepressant responders and non-responders included in PBMCs protein expression analysis

Characteristic	Responders<br>Mean (± SD)	Non-responders<br>Mean (± SD)	Statistics
Age	47.1 (16.1)	51.5 (16.1)	*p* = 1.0
Males/Females	5/10	6/11	χ2 = 0.136, p = 0.91
HDRS at admittance	23.4 (5.3)	22.5 (4.2)	*p* = 0.52
HDRS at T6	4.6 (3.7)	18.9 (5.2)	*p* < 0.0001

HDRS, Hamilton Depression Rating Score
T6, after six weeks of admission

Supplementary Table S2. Demograhic and clinical characteristics of antidepressant responders and non-responders included in ex vivo PBMCs cultivation and paroxetine treatment.
